# Identifying and addressing barriers and opportunities for bovine respiratory disease complex vaccination: a consensus paper on practical recommendations for best practise vaccination

**DOI:** 10.3389/fvets.2024.1368060

**Published:** 2024-04-05

**Authors:** Matt Yarnall, Federica Amovilli, Sébastien Assié, Jade Bokma, Matt Pugh, Dirk Werling

**Affiliations:** ^1^Boehringer Ingelheim Vetmedica GmbH, Ingelheim, Germany; ^2^Via A. Reverberi, Montecchio Emilia, Italy; ^3^Oniris, Veterinary School Route de Gachet Cedex, Nantes, France; ^4^Faculty of Veterinary Medicine, Department of Internal Medicine, Reproduction and Population Medicine, Ghent University, Ghent, Belgium; ^5^Belmont Farm and Equine Veterinarians Ltd., Rotherwas, United Kingdom; ^6^Centre for Vaccinology and Regenerative Medicine, Department of Pathobiology and Population Sciences, Royal Veterinary College, London, United Kingdom

**Keywords:** BRDC, vaccination, barriers, opportunities, systemic, intranasal

## Abstract

Many questions are raised, and challenges faced in the new era of (intranasal) bovine respiratory disease complex vaccination. An increase in vaccination rate is expected, due to its positive impact on cattle health, reduction of antimicrobial use and economic factors. However, engagement of farmers and veterinarians with regard to vaccination is often affected by limitations, resulting in the development of barriers to vaccination, but also opportunities to overcome these. The objective of the report is to provide practical recommendations and a consensus on best practises for BRDC vaccination, addressing barriers faced by veterinarians and farmers. The report combines an evidence review with expert opinions and includes discussions on different vaccination approaches, such as intranasal and systemic protocols. As result of the discussions, several barriers to BRDC vaccination were identified, including a lack of knowledge or visibility of the disease’s impact, the preference for blanket antibiotic use over vaccination, resistance to change, the need for visible success, uncertainty about the best time to vaccinate, and concerns about adverse reactions and vaccine efficacy in the presence of maternal antibodies. While these barriers seem substantial, they provide opportunities for the veterinary sector. Indeed, veterinarians are encouraged to use the argumentation presented, along with local case studies and diagnostic testing to highlight the impact of disease, while conducting calf health audits, ensuring expectations are managed to achieve visible success. Overall, this consensus paper aims to provide practical recommendations and support for veterinarians and farmers to overcome barriers and increase BRDC vaccination rates in cattle.

## 1 Introduction

Bovine respiratory disease complex (BRDC) is the biggest endemic cause of morbidity and mortality in growing cattle in production systems worldwide (1). It results in significant demand for antimicrobial use (2), significant economic impact, decreased well-being (3) and increased CO2 emissions per litre of milk or kilo of meat produced (4). Prevention of BRDC is the ultimate goal, through improved cattle resilience, housing and management.

In addition to optimal calf management, cattle resilience can be increased through vaccination against BRDC-causing pathogens. Current BRDC vaccination rates vary across Europe and between causative pathogens, with vaccination rates of 45% for bovine viral diarrhoea (BVD) and 22% for infectious bovine rhinotracheitis (IBR) in the United Kingdom (5). There is a trend for increasing vaccination rates, with the proportion of cattle vaccinated against BRDC changing from 29% in 2011 to 44% in 2021 (6). However, in many regions there is a clear need to increase this further, and despite many opportunities to do so, there are also many—perceived and real—barriers.

Veterinarians across Europe are looking for support to overcome these barriers, including a consensus on best practise with regards to vaccination schedules against BRDC. This is currently lacking in the literature. The needs of farmers for BRDC prevention under varying situations, combined with the challenge of aligning with data sheet recommendations, do not always ensure that cattle receive optimal protection.

## 2 Objectives

This extensive evidence-review and expert opinion paper aims to provide practical recommendations to motivate farmers to vaccinate against BRDC and a consensus on best practise BRDC vaccination to ensure veterinarians can advise their farmers with confidence.

## 3 Materials and methods

The consensus presented here is a result of a multilayer approach.

Initially, a workshop was held in Lisbon in March 2023 to establish the—perceived or real—barriers to, and benefits of, vaccination against BRDC. 41 delegates attended, consisting of animal health professionals from across Europe, representing veterinary technical managers, marketers, and sales representatives of a leading animal health company. The context of different BRDC vaccination approaches, namely intranasal, systemic and combined protocols, were introduced by two practising cattle veterinarians from the United Kingdom and Italy, and by a BRDC epidemiologist joining remotely from France.

Following the establishment of the barriers and benefits of BRDC vaccination from the customers’ perspective, a series of online discussion sessions were held subsequently between March and June 2023 with a group of five European experts. These sessions sought to provide the evidence base to support the benefits and overcome the barriers to vaccination.

The following sections provide the reasoning to overcome the barriers identified combined with practical recommendations, which are broken down into general, intranasal and systemic vaccination barriers. This is then followed by the benefits and/or opportunities identified to motivate veterinarians and their farmers to increase BRDC vaccination rates, along with reasoning to overcome the barriers identified and practical recommendations.

## 4 Results

### 4.1 Identified perceived or real barriers

As a result of the workshop held, the following perceived or real barriers to BRDC vaccination were identified (Table 1). These are addressed in the following sections.

**Table 1:** Veterinarians and farmer combined barriers to BRDC vaccination in general, and specifically to intranasal and systemic vaccination.

BRDC vaccination barriers	Intranasal vaccination barriers	Systemic vaccination barriers
Lack of visibility of the problem and reluctance to spend money	Perception of loss of vaccine out of nose	Maternally derived antibody (MDA) interference impacting efficacy
Blanket antibiotics are easier for farmers	Use of a nozzle	Sub-cutaneous administration
Resistance to change and time investment needed with advice and vaccination	Need for mixing	Longer onset of immunity (OOI)
Need for visibility of success	Shorter duration of immunity (DOI)	Welfare issues of needle use
Is follow up vaccination needed?	When is too early to vaccinate?	Two injections needed
Does vaccination of a stressed animal lead to disease?		
Practicality		
Potential adverse reactions and decrease in milk production if vaccinating milking herd		
Concurrent use with other vaccines		
Unsure of the best time to vaccinate		
Vaccination does not cover all potential pathogens		
Compliance of storage and use		

Humans by their nature are resistant to change, and farmers are no different. Time is needed to understand the motivations and overcome the barriers of farmers, and this can be an opportunity to provide health planning services on farm to help reduce disease and drive increased service fees, replacing margin on antibiotic sales.

Veterinarians often face the challenge to motivate farmers to vaccinate due to the insidious nature of an endemic disease or due to the risk of an epidemic in a naïve herd. Farmers are motivated to treat disease when it is visible, and the benefits are often clear to see. Also, it can seem cheaper to the farmer, to treat some or all the animals with antibiotics, rather than vaccinating all animals. However, farmers often miss the hidden impact of disease, indeed a United States study looking at 469 steers at slaughter showed that 72% had lung lesions, but only 35% had been treated. Those calves with lesions had 76 g/day less average daily gain (ADG) (7).

Similarly, in a European study assessing 956 calves, approximately 20% showed clinical signs of BRDC, while 42.8% had lung lesions (8). A further study assessing the association between weight at slaughter and earlier lung lesions (calves with uncured or chronic pneumonia) showed a lower ADG (992 ± 174 and 930 ± 146 g/day, respectively) compared with calves that never developed pneumonia (ADG = 1,103 ± 156 g/day) (9).

From these studies it is clear that a reduction of BRDC has a positive production impact; however, another impact is the reduction of treatment with antimicrobials. Industry is moving to reduce use of antimicrobials to decrease the generation of resistant pathogens (2, 10). Several EU countries offer financial incentives to farmers who show that they are decreasing antimicrobial use. Italy, for instance, has developed a programme called “ClassyFarm” (10), a platform that collects data on a number of variables relating to antimicrobial use (and other critical points such as animal welfare) and produces a “score” for each farm. Using a BRDC vaccine contributes to a better ClassyFarm “score,” and greater financial support.

Expectations should be managed to achieve visible success and a return on the investment made, which can motivate farmers to vaccinate and continue vaccinating. This is best done in partnership with the farmer, built around understanding of risk and how to work collectively to best mitigate this. Various tools including vaccination and monitoring can be used to enable ongoing review and support vaccination over multiple seasons. The veterinarian can also use local case studies, cost benefit analyses and lab or calf-side diagnostic testing to highlight the presence and impact of disease.

Often BRDC vaccination is performed in the face of disease or in a high-stress situation, such as before or after transport. Animals vaccinated when healthy may already be harbouring infection, and therefore succumb to infection shortly after vaccination, before immunity has been established. In this situation it is critical to ensure vaccination has been performed before the risk period to allow time for immunity to develop.

A commonly perceived challenge is the time and effort required for vaccination, especially with globally relevant issues such as lack of labour. This is further exacerbated by handling of larger beef animals. The main consideration here is that a one-off planned vaccination effort to prevent disease is less effort than to treat animals individually.

If vaccination of the milking herd in addition to youngstock is undertaken, to reduce circulation of pathogens and disease incidence, risk of impact on milk yield has been raised as a concern. However, all vaccines go through stringent testing to demonstrate a positive risk–benefit analysis, and this really needs to be emphasised. This includes overdosing and repeated dosing and monitoring the frequency of adverse reactions (e.g., fever or nasal discharge). The results are indicated on the product data sheet. However, it is also extremely important to re-iterate that the effects seen as a result of vaccination are negligible compared to those happening after a real infection.

If the farmer feels that the risk of potential reactions and possible impact on production is not balanced by the gain in protection of the herd, it may be beneficial to consider vaccination of cows during the dry period. This “cocooning effect” for the neonates has the added benefit of stimulating a boost in specific BRDC antibodies in colostrum (11). However, the dry period can be a busy time for vaccinations, and while there is rarely data on use of more than one vaccine at once in cattle, it is actively encouraged for multiple antigens to be given at the same time in children. For example, babies in the United Kingdom receive a 6-in-1 systemic vaccine at 8 weeks of age, along with an oral rotavirus vaccine and systemic meningitis B vaccination (12). Cattle are exposed to many pathogens at any one time so from an immunological perspective the immune system is constantly being challenged. Best practise would be to only vaccinate healthy animals and on alternate sides of the animal to stimulate opposite lymph nodes.

It can be a challenge to know the best time to vaccinate, based on age, risk period or seasonality. In general, to acquire a reduction in shedding it is best to vaccinate all year round, based on age. This can then be boosted or prioritised before periods of increased risk.

While vaccination against all pathogens is not possible, it is better to limit impact of as many pathogens as possible to stop one primary infection predisposing to a secondary infection. There can also be a stimulation of a “generalised” local, and subsequent systemic immune response from the use of intranasal vaccination, mediated through interferon (IFN), either through the antigen or the adjuvant, which helps provide immediate protection against other respiratory pathogens in the airspace (13).

With regards to vaccine storage and use compliance, veterinarians can support excellent compliance through provision of cool bags, fridge thermometers and vaccination devices.

#### 4.1.1 Barriers to intranasal vaccination

Farmers can be frustrated by nasal leakage of intranasal vaccine. Use of a nozzle can reduce this, ensuring appropriate particle size to maximise distribution and mucosal immunity stimulation. Work has shown this is best with the head at a 45° angle (Glenn, Berner, Bomphrey, Werling, unpublished observation), with the dose split between both nostrils in case one is blocked or full of mucous, and to ensure maximal mucosal surface exposure and response. Use of a nozzle also aids splitting the dose, as squirting one half of a 2 mL dose into the nostril at pressure can be challenging, with a tendency to either use too little pressure or release the whole dose. Furthermore, provision of mixing spikes by veterinarians, and advocating mixing in a batch at the start of each working session can minimise the challenge of preparing live intranasal vaccines.

The duration of immunity (DOI) as demonstrated by challenge is shorter for intranasal compared to systemic vaccination. However there is an increasing awareness of “trained immunity,” which defines the ability of the innate immune system to form immune memory and provide long-lasting protection against foreign invaders. In general, trained immunity is known to provide relatively short-term protection ranging from about 3 months to 1 year (14).

Some intranasal vaccines suffer from interference with maternally derived antibodies (MDA). Martinez et al., showed that modified-live virus (MLV) BRSV vaccines reduced the risk of morbidity in calves with absence of MDA at initial vaccination, but failed to demonstrate significant morbidity reduction when calves were vaccinated in the face of MDA (15).

A further study demonstrated preferential weight gain, compared with unvaccinated calves, for those intranasally vaccinated at a slightly older age than those at a younger age. The authors suggested that younger calves may have been less able to respond to the vaccination due to having an immature adaptive immune system, or inhibitory effects of maternal antibody (16).

With regards to the mechanism of this potential inhibitory effect, tight junctions lining the mucosa are still relatively open at Day 0 and are only closed after day 1 to 2. This is most recognised in the gut but it would follow that the respiratory mucosa would act similarly (17, 18). Combined with the fact that IgG (via the neonatal Fc receptor) and IgA are actively transported across the nasal mucosa, although in hugely different amounts, both mechanisms could result in interference with intranasal vaccination (17, 19). This would be particularly relevant for calves vaccinated less than 5–10 days old as the half-life of serum IgA is 2.5 days (19).

It is therefore important to note that some vaccines are unique in that they suffer no interference from MDA, demonstrating efficacy through challenge with and without presence of MDA (Bovalto® Respi Intranasal) (20, 21). Whereas no assurance of efficacy can be guaranteed with other intranasal vaccines in presence of MDA (21). In addition, some vaccines that may be used at a very young age are associated with a higher risk of adverse reactions such as fever which could inhibit feeding or divert energy for growth to energy to mount an immune response, which would be detrimental at this very early age (22).

#### 4.1.2 Barriers to systemic vaccination

While some intranasal vaccines can achieve full efficacy in the presence of MDA, systemic vaccination is more likely to experience interference from MDA (23). If systemic vaccination is required in a younger calf, the second vaccination should be no sooner than the recommended re-vaccination window. A follow up booster may be advocated before the limit of the DOI to ensure the required protection is achieved.

If a shorter onset of immunity (OOI) is required, intranasal vaccination can be undertaken in addition to systemic vaccination, taking advantage of the Prime-Boost effect in those vaccines containing the same antigens in live intranasal and killed systemic form. The need for two vaccinations with most systemic vaccines ensures longer DOI following an anamnestic response, which can be further primed with a specific intranasal vaccine.

Vaccines should be administered with sharp, clean needles, and vaccination devices that sterilise the needle between applications are not recommended for live vaccines due to the risk of inactivation. In some markets, intramuscular vaccination is not recommended due to injection site reactions and/or damage to meat at inspection.

### 4.2 Opportunities to overcome these barriers

Veterinarians can motivate their farmers to increase BRDC vaccination through use of the following benefits, as listed in Table 2. These are discussed in the following sections.

**Table 2:** Vet and farmer combined opportunities for BRDC vaccination in general, and specifically to intranasal and systemic vaccination and in combination.

BRDC vaccination opportunities	Intranasal vaccination opportunities	Systemic vaccination opportunities	Combination vaccination opportunities
Decrease antibiotic usage/Decrease vet and or treatment costs	Use at early age	Longer DOI	Start with IN, continue with injectable (Prime-Boost)
Decrease morbidity/mortality	Potential for use in presence of MDA	Broader protection	Vaccinate older animals for cocooning effect and boosting colostrum
More productive and resilient calves protecting the future potential of herd	Rapid OOI	Easier administration	Vet and farmer engagement on bespoke protocols
More opportunity to be proactive vs. reactive	Can use alongside treatments	No shedding of vaccine strains	
Decrease virus load in herd	Single dose	Requirement for two doses	
	Easier administration in younger animals		

#### 4.2.1 General identified opportunities to overcome vaccination barriers

As stated by O’Connor et al., vaccination against causal organisms is a frequently used and preferred approach to controlling BRDC because it reduces the need for antibiotic use and improves animal well-being (24). However, while reductions in morbidity and mortality have been clearly observed following vaccination against pathogens involved in BRDC (25, 26), data demonstrating reduction in antibiotic use is more elusive, and are even difficult to obtain for human studies (27). Nevertheless, there are published data comparing vaccination protocols demonstrating decreased number of cattle requiring second and third antimicrobial treatments for clinical BRDC, thus demonstrating the value in vaccination for reduction of antimicrobial use (28). In addition, another paper demonstrated significantly fewer treatments for BRDC in calves vaccinated with a combined viral and bacterial vaccine (10.8%) than calves vaccinated with purely viral components (18.1%, *p* = 0.04) (29).

Looking at the impact of morbidity and mortality, a recent systematic review and meta-analysis was performed on experimental challenge studies with BRSV in non-vaccinated or vaccinated calves. The analysis demonstrated a 41.3% reduction in the mortality risk for vaccinates, compared with controls, independently of type of vaccine used. A 50.6% reduction of the morbidity risk was also demonstrated in trials in which calves had no MDA at initial vaccination (30). These findings are not only true for viral agents of BRDC, but also for bacterial pathogens. A published study of a vaccine registration demonstrated a significant reduction of clinical signs and lung lesions following a *Mannheimia haemolytica* challenge in vaccinated animals versus controls (31).

When looking at natural exposure trials, beef calves vaccinated with various antigen combinations had a significantly lower BRDC morbidity risk than the non-vaccinated control calves (25). In addition, vaccination of beef calves around the time of weaning with multivalent MLV vaccines alone or in combination with *M. haemolytica/P. multocida* bacterins reduced BRDC morbidity and mortality after weaning (32).

Thus, the authors believe that especially in the area of beef calf rearing, substantially more impact on the occurrence of BRDC could be achieved by vaccinating animals on the producers’ farms, before shipment, even if this may only lead to an “unspecific” stimulation and modulation of the innate immune system. The quickest way to achieve this is by vaccinating calves intranasally. Indeed, regarding intranasal vaccination against PI3, BRSV and IBR, Olivett et al. demonstrated that while an intranasal vaccination protocol did not affect the occurrence of abnormal respiratory scores, it did reduce the risk of lung lesions associated with pneumonia (33).

Vaccination for BRDC has been shown to result in more productive and resilient calves. In one calf unit, 497 calves (mean age 19 days) were included in a study, 247 of which were vaccinated at the time of arrival to the unit and 250 served as negative controls (non-vaccinated). Intranasal vaccination combined with older arrival age (17 days or older) resulted in a higher daily gain (47.8 g/day) compared with non-vaccinated calves (*p* = 0.003) (16). It is worth highlighting that some of these productivity benefits of vaccination may be due to the impact on subclinical disease, as described earlier (7, 9).

Most BRDC vaccines are licenced to reduce shedding, and the benefits of reduction of virus shedding are a reduction in infection pressure, which can protect those animals that may be more vulnerable. Indeed, a whole herd vaccination programme can foster a herd-immunity, resulting in a “cocooning” effect on newborn calves, meaning these are protected as every individual in their vicinity is protected, and thus reducing pathogen pressure on neonatal calves (11). Vaccination during the dry period can also be used to stimulate passive transfer, to the calf.

Thus, veterinary support is key to ensure optimal vaccination strategies. This is also relevant with regards to compliance, considering studies showing that for example farmers only vaccinated at the right time with the second dose of a vaccine course 48% of the time (34). It is also relevant with regards to potentially complicated vaccination schedules. Lava et al. highlighted that despite the use of BRDC vaccines significantly decreasing mortality risk, continuous stocking of calves and high age variability results in suboptimal vaccination management (35).

In addition to the points discussed above, one has to keep in mind that every activation of the immune system requires energy. Indeed, an increase of the body temperature by 1°C requires a basal energy turnover increase of about 30% (36). Thus, it is clear from these data that there are productivity and thus economic benefits to decreasing BRDC incidence.

For many veterinary practitioners, vaccine sales and the ability to sell a calf health service are a key part of practise income. One of the authors notes that income from vaccine and services grew at 10.6% and 8.2%, respectively, in 2022 vs. 2021, compared to 2.6% on antibiotic sales growth. The service element of this is key to demonstrating to farmers the success of the shared partnership, i.e., reduced antibiotic usage, health and performance gains.

#### 4.2.2 Opportunities to overcome barriers to intranasal vaccination

Intranasal vaccination provides the benefit of use at an early age, making use of the calf’s fully functional innate immune system from birth. Calves possess a high amount of a specific T cell subset, the gamma delta T cells which have been shown to combine innate and adaptive functions, but more importantly regulate immune responses from beneath the mucosal surfaces (37). The stimulation of local, mucosal immunity does not require a fully functioning adaptive immune system to process a systemic vaccine (38). There is also less risk of interference with MDA, albeit this must be caveated depending on vaccine and age of use. Thus, antibodies present in the blood from colostrum provide systemic protection and the mucosal surfaces of the calf are protected by the components of the innate immune system further boosted by the intranasal vaccination. This concept is further enhanced by the notion of “trained immunity” (14). The intranasal application of vaccines may subsequently also stimulate the “common mucosal system.” This concept explains the situation where lymphocytes induced by an antigen in a mucosal site migrate to other mucosal sites as effector cells to protect all mucosal tissues from the same antigen. Hence, upper respiratory tract mucosal immune sites are one of the targets for mucosal vaccination to combat mucosal infectious diseases and to induce respiratory immune protection.

The rapid OOI provided by intranasal vaccination is afforded by the stimulation of the components of the local innate immune responses comprised, amongst other cells, of granulocytes, monocytes/macrophages, dendritic cells, gamma delta T cells and natural killer cells (NK cells) (39, 40).

The mucosal response can be specific, against primarily PI3 and BRSV, but also provides a generalised stimulation of the local innate immune response mediated by IFN (13). In addition, the space-occupying effect also reduces the risk of population by other pathogens. This is also of benefit in an outbreak situation, allowing a more rapid immune response without the same demands on the immune system as systemic vaccination (15). Aligned with this benefit, intranasal vaccination can also be used alongside treatments if required, due to the lack of interference with the systemic immune response. While it is clear that both nostrils need to obtain a vaccination dose to stimulate mucosal immune responses in both nostrils, it is not yet completely clear for cattle whether mucosal IgA levels can be boosted by a second intranasal vaccination. However, it is becoming clear that systemic booster vaccination also increases mucosal IgA responses in fully immune-competent calves (41).

Intranasal vaccination should also be at reduced risk of interference by a stress response, which can often accompany the transport and handling of youngstock (42). This is also complemented by the need for just one vaccination, allowing for minimal handling and increased compliance when receiving or moving stock, particularly when used with appropriate facilities to allow access to the head. Intranasal vaccination may also be of benefit in some markets due to lack of potential injection site reaction caused by SC or IM vaccination.

#### 4.2.3 Opportunities to overcome barriers to systemic vaccination

The DOI of systemic vaccines is variable depending on the vaccine formulation but also on the ratio of MDA to vaccine antigens at the time of vaccination (43). The DOI is generally longer with systemic vaccines than with intranasal vaccines when looking at marketing authorisations and published studies (44, 45), but it is worth checking the DOI as demonstrated by challenge studies. Protection provided by systemic vaccination covers not just viral antigens, but also bacterial pathogens, which are more likely to lead to secondary and systemic impacts. In addition, the breadth of the immune response achieved through systemic vaccination following stimulation of the mucosal response is noted below with regards to Prime-Boost vaccination. For some farm systems, particularly for older animals or where head restraints are not available, systemic vaccination is also preferred for ease of use and safety of the user. Also, these vaccines are often ready to use, resulting in shorter preparation time. Shedding of vaccine strains can occur following use of a live vaccine, however there is no possibility of vaccine organisms spreading between animals following administration of a killed vaccine. It should be taken into account that viral strains following MLV BRSV intranasal vaccination are easily detected by PCR on nasal swabs several days after vaccination (46). This may therefore interfere with the detection of pathogens in the event of a disease outbreak but cannot transmit the disease to other animals. This may be of more relevance following use of live IBR vaccines when animals are destined for IBR restricted establishments.

Lastly for this section, in some markets where veterinarians perform vaccination, a systemic vaccine two dose primary course is another opportunity to review calf health on farm.

#### 4.2.4 Opportunities to overcome barriers to combination vaccination

The flexibility of intranasal and systemic vaccination can be utilised to provide a rapid OOI, combined with the duration and breadth of protection elicited by systemic vaccination. The benefits of a heterologous Prime-Boost approach can be utilised, using the same antigens in live (intranasal) and inactivated (systemic) form, allowing for a rapid anamnestic response, utilising cellular and humoral immunity for broad and prolonged protection (47). This has also been demonstrated by Erickson et al. who compared three different vaccination protocols in 75 beef calves. The calves vaccinated at an early age with intranasal and then boosted with a killed vaccine at 48 days had a clear increase in antibodies post-turnout vaccination, compared to live vaccine boosted calves (48). To achieve the best results from vaccination, a bespoke protocol tailored to the farm situation is required to understand how the cattle are managed within it and what risks they are exposed to. This is a great opportunity to manage risk and expectations and achieve success through demonstrating a return on the investment made.

A BRDC audit is essential and should include a review of:

- Colostrum management: Impacting health and immunity of young calves and affecting use of intranasal versus systemic vaccination. There may also be a consideration of the use of cocooning (11).
- Housing/environment: This will impact infection pressure and may affect consideration of whole herd and all year round versus seasonal vaccination.
- Age of animal: Age of animal will affect decision of ease of use and efficacy of intranasal versus systemic vaccination.
- Diagnostics: This can inform the most appropriate vaccination routine.
- Key performance indicators: These will inform what measurements are taken and can be used to motivate, by demonstrating success and ultimately a return on investment.

## 5 Conclusion

This consensus paper aims to provide practical recommendations and support for veterinarians and farmers to overcome barriers and increase BRDC vaccination rates. The paper identifies several barriers to BRDC vaccination, including a lack of knowledge or visibility of the disease’s impact, the preference for blanket antibiotic use over vaccination, resistance to change, the need for visible success, uncertainty about the best time to vaccinate, concerns about adverse reactions and vaccine efficacy in the presence of maternal antibodies. To overcome these barriers, the paper provides evidence-based logic to overcome them, and also identifies opportunities to further drive increased prevention of BRDC through vaccination, as opposed to treatment of BRDC using antibiotics.

## Data availability statement

The raw data supporting the conclusions of this article will be made available by the authors, without undue reservation.

## Ethics statement

The authors confirm that the ethical policies of the journal, as noted on the journal’s author guidelines page, have been adhered to. No ethical approval was required as this is a review article with no original research data.

## Author contributions

MY: Conceptualization, Investigation, Resources, Writing – original draft, Writing – review & editing. FA: Conceptualization, Formal analysis, Writing – original draft, Writing – review & editing. SA: Conceptualization, Formal analysis, Writing – original draft, Writing – review & editing. JB: Conceptualization, Formal analysis, Writing – original draft, Writing – review & editing. MP: Conceptualization, Formal analysis, Writing – original draft, Writing – review & editing. DW: Resources, Validation, Writing – original draft, Writing – review & editing.
